# Bacteriology

**Published:** 1904-11-12

**Authors:** 


### PROGRESS IN MEDICINE AND SURGERY.

### BACTERIOLOGY.

**The Leishman-Donovan Body.**—The discussion at the recent meeting of the British Medical Association[1] on this interesting parasite may be summarised as follows:—Cases of infection by the Leishman-Donovan body have been reported from India, China, Tunis, Algiers, Arabia, and Egypt. The parasite is found in the spleen, liver, mesenteric glands, kidney, and in intestinal ulcers. It is met with inside large mononuclear cells, and the so-called "stroma" in which masses of the parasite have been found is in all probability the disintegrated groundwork of such cells. Leishman believed the organism to represent a stage in the life-history of a flagellate organism closely resembling a trypanosoma, if not actually belonging to that genus. Involution forms of trypanosomes appear in sections of tissues exactly like the Leishman-Donovan bodies, and Schaudin and Prowazek have seen trypanosomes infecting owls and house-flies go through non-flagellated resting-stages. From the frequent association of the disease with intestinal ulceration it is possible that the parasites are eliminated by the bowel. Rogers examined a number of cases of cachexial fever, and found the Leishman-Donovan body present in the blood obtained by spleen puncture. Bacteriological examinations gave negative results. If, however, a little of the blood were added to a small quantity of sterile citrate of soda solution to prevent clotting, and then kept in the cold incubator at 22° C., the parasites multiplied rapidly, and, after some days, there developed from the bodies typical trypanosoma with thick flagella and macro- and micro-nucleus. He was able to grow trypanosoma in this way from cases of Kala-azar and cachexial fever. Bentley, who has studied the parasite in Assam stated that fish living in waters which are polluted by the sewage of districts infected with Kala-azar, are found to contain trypanosomes. He thinks these bodies may prove to be the spores of some micro- or myxo-sporidian. Christopher says that the cycle of development is as follows:—The forms divide by binary fission, the parasites enter an endothelial cell, proliferate, burst the cell, and spread in the blood plasma. The disease is a systemic infection of the septicæmic type. Low stated that, in contradistinction to Rogers' results, he had failed to get any development from bodies kept at room temperature in hæmoglobin agar or hæmoglobin broth, nor did any change take place in fresh specimens ringed with vaseline and watched under the microscope for four days.

**Typhoid Fever.**—A research by Rolly[2] confirms the fact that the bacillus typhosus may be detected in the blood of persons suffering from enteric fever, and that the procedure of sucking up blood into capillary tubes for the purpose of making Widal's test is not devoid of risk. He drew off 20 ccs. of blood and mixed this with melted glycerine agar, and then incubated. In 88 per cent. of 50 cases, typhoid bacilli developed, and the colonies were generally very numerous. The bacilli were most numerous in the acute stage, and were not detected after the subsidence of the fever. The number of bacilli bore no relation to the severity of the case, and they were sometimes found before Widal's reaction was obtained. To obviate the risk of sucking up the blood Jones[3] suggests that after pricking the ear the blood be collected in the white counter of a Thoma-Ziess hæmocytometer, and that this be diluted in the ordinary way by adding ten times the volume of distilled water. The broth culture of typhoid bacilli should, he says, be drawn up into a graduated pipette by means of the screw-compressing pipette of Nabarro's type. Whilst these measures would appear to insure greater security, it should be remembered that the instruments would require careful sterilisation. Max Einhorn[4] has treated ten cases of typhoid fever with a serum prepared by Tavel and Jez of Berne. Daily doses of 6 to 12 cubic centimetres were given, treatment commencing from the ninth to the fifteenth day and being continued until the temperature remained under 102° Fahr. The fever did not appear to be shortened, but after each injection the temperature was slightly lowered and the general symptoms, especially those referred to the nervous system, were markedly alleviated. No ill effects followed the injections, and he anticipates that with more potent sera this treatment will meet with general approval.

**Emphysematous Gangrene.**—Dudgeon and Sargent[5] report a case of emphysematous gangrene following a compound fracture of the leg. The bacteriological examination showed in fresh films, and in ærobic cultures a coccus which was staphylococcus albus. Anærobic cultures from the wound exudate showed a growth of bacillus coli, and the identity of the organism was confirmed by Marmorek's test and by subcultures. Cultures from the patient's blood gave negative results. The bacillus was pathogenic to guinea-pigs, but no gas formation occurred; in this particular their results agreed with those of Welch, Klein, and Sanfelice. Chavigny by inoculating his bacillus together with staphylococcus aureus succeeded in producing emphysema, but the present writer did not succeed with mixed cultures. Welch claims to have produced emphysema of the tissues by the intravenous injection of B. capsulatus ærogenes, and Corner and Singer by injections with B. œdemates aërobius. It seems probable that many of the bacteria described in association with emphysematous gangrene are varieties of B. coli, and that insufficient care has been taken to ensure their identification.

**Bacterial Air Pollution.**—Gordon[6] has repeated Flügge's experiments for determining the number and variety of bacteria in human breath. He artificially infected the mouth with B. prodigiosus, and placing culture plates at certain distances he noted the degree to which they were infected. During continued loud speaking he found that bacteria were projected for a distance of 40 feet in front and 12 feet behind the speaker. The organisms most frequently found in droplets of saliva coming from the mouths of healthy persons were the streptococcus brevis, medius, longus, and conglomeratus, and of these the brevis could be detected in ten-millionth to one hundred-millionth part of a cubic centimetre of saliva. The streptococci of the air could, he states, be easily distinguished from those obtained from human saliva, and these last speedily die off in the open air.

**Action of Micro-organisms in Cæcal Digestion.**—Macewen,[7] in the course of his Huxley lecture, states that bacteria flourish in the cæcum and appendix, aud that there they break up the food particles that have resisted the action of the digestive juices. The secretion from the appendix stimulates and controls the action of the bacteria and prevents them injuriously acting upon their host, being assisted in this work by the leucocytes in the solitary lymph follicles and by the nuclein. The cell walls of the appendix are possibly storehouses of germs which are poured out when needed. If the functional power and vitality of the appendix be lowered the germs may be capable of damaging the coats of the cæcum. Lauz and Tavel[8] have made a bacteriological examination in 138 cases of appendicitis. They state that the normal appendix is never sterile, there being a variety of organisms present, of which the B. coli is the commonest. Ten per cent. of appendices pathologically affected were sterile; 37·5 of cases of cold abscess in the appendix were sterile, and in the remainder only a few forms were found; 75 per cent. of cold extra appendicular abscesses were sterile. Acute perityphlitic abscesses are always microbic. The absence of bacteria in old pathologically affected appendices is, in the author's opinion, due to active phagocytosis, and when the appendix has recovered the leucocytes are withdrawn and the remaining fluid is sterile.

[1] Brit. Med. Jour., Sept. 17, 1904. [2] Munch. Med. Woch., June 14, 1904. [3] Clin. Jour., July 6, 1904. [4] Med. Record, N.Y., Jan. 16, 1904. [5] Trans. Path. Soc., vol. 35, part ii., 1904. [6] Rep. Med. Officers, L.G.B., 1902–1903. [7] Brit. Med. Jour., Oct. 8, 1904. [8] Rev. de Chir., 7, 8, 1904.

